# 局部晚期非小细胞肺癌同步放化疗中紫杉醇两种用法的多中心、随机对照研究

**DOI:** 10.3779/j.issn.1009-3419.2011.03.08

**Published:** 2011-03-20

**Authors:** 安辉 石, 广迎 朱, 荣 余, 颖杰 王, 廷毅 夏

**Affiliations:** 1 100142 北京，北京大学临床肿瘤学院、北京肿瘤医院暨北京市肿瘤防治研究所放射治疗科，恶性肿瘤发病机制及转化研究教育部重点实验室 Department of Radiation Oncology, Beijing Cancer Hospital and Institute, Peking University School of Oncology, Beijing 100142, China; 2 100142 北京，空军总医院放射治疗科 Department of Radiation Oncology, Air Force General Hospital, Beijing 100142, China

**Keywords:** 肺肿瘤, 综合疗法, 放射疗法, 紫杉醇, Lung neoplasms, Combined modality therapy, Conformal radiotherapy, Paclitaxel

## Abstract

**背景与目的:**

尽管美国国立综合癌症网络推荐同步放化疗为不可手术局部晚期非小细胞肺癌的标准治疗手段，但同步放化疗方案仍有争议，本研究旨在比较适形或调强放疗合并紫杉醇每周三次用法和相同总剂量的每周一次用法治疗局部晚期非小细胞肺癌的优劣，以优选放疗中紫杉醇的最佳给药方案。

**方法:**

2006年4月-2009年4月，全国多中心顺序入选符合条件的局部晚期非小细胞肺癌患者，采用随机数字表法将入组患者随机分为两组，放疗要求95%PTV达（60-70）Gy/（30-35）次/（6-7）周。

**结果:**

共38例患者入组，每周三次用法组18例，1例治疗结束后1年出现3度放射性肺损伤，后因肺内感染呼吸衰竭死亡，3例患者出现1度-2度放射性肺炎，1例患者出现3度放射性食道炎，12例患者出现1度-2度放射性食道炎，13例患者出现1度-2度白细胞减少；每周一次用法组20例，3例患者出现3度放射性肺炎，3例患者出现2度放射性肺炎，1例患者出现3度放射性食道炎，10例患者出现1度-2度放射性食道炎，1例患者出现4度白细胞减少，14例患者出现1度-3度白细胞减少。两组患者有效率分别为88.8%和50.0%（*P*=0.026）。两组患者1年生存率分别为79%和67%（*P*=0.607）。

**结论:**

适形或调强放疗合并紫杉醇每周三次用法较每周一次用法治疗局部晚期非小细胞肺癌安全、有效。

## Abstract (en)

**Background and objective:**

Although the guidelines of the National Comprehensive Cancer Network of USA recommend that the standard therapy for locally advanced non-small cell lung cancer (LANSCLC) is concurrent chemoradiotherapy. There is ongoing controversy about the treatment regimen which combines chemotherapy concurrently with radiotherapy. The aim of this study is to compare weekly usage with triple weekly usage of paclitaxel in concurrent radiochemotherapy for patients with LANSCLC, and to obtain the best paclitaxel regimen in the concurrent radiochemotherapy.

**Methods:**

From April 2006 to April 2009, some LANSCLC patients in multicenter were randomly divided into weekly usage (45mg/m^2^, 1 times/week, a total of 270 mg/m^2^ in six weeks) and triple weekly usage (15 mg/m^2^, 3 times/week, a total of 270 mg/m^2^ in six weeks) group of paclitaxel by a random number table. All patients were treated with 3D radiotherapy, and 95% planning target volume (PTV) received a prescription dose of (60-70) Gy/(30-35)times/(6-7)weeks, (1.8-2.0) Gy/fraction. Then the side effects, response and overall survival rate were compared between two groups of patients.

**Results:**

Thirty-eight LANSCLC patients were enrolled. Weekly usage and triple weekly usage group were 20 and 18 patients, respectively. In the triple weekly usage group, the side effects were 12 patients had radiation esophagitis of Ⅰ-Ⅱ degree, 1 patient had radiation esophagitis of Ⅲ degree, 2 patients had radiation pneumonitis of I degree, 1 patient had radiation pneumonitis of Ⅱ degree, 1 patient had radiation pneumonitis of Ⅲ degree and died of respiratory failure, 2 patients developed weight loss of I degree. In the weekly usage group, the side effects were 11 patients had radiation esophagitis of Ⅰ-Ⅲ degree, 6 patients had radiation pneumonitis of Ⅱ-Ⅲ degree, 2 patients developed weight loss of I degree, 6 patients developed leucopenia of Ⅲ-Ⅳ degree. The response rate of two groups was 88.8% and 50.0%, respectively (*P*=0.026). 1-year survival rate of two groups was 79% and 67%, respectively (*P*=0.607).

**Conclusion:**

Although the preliminary results did not show the merits of survival in triple weekly usage, but preliminary results show that triple weekly usage was more safe and effective than weekly usage of paclitaxel in concurrent radiochemotherapy for patients with LANSCLC.

我国肺癌中70%-80%为不能手术的中、晚期患者，这类患者的治疗是国内外肿瘤临床研究的热点之一。近20年，RTOG 8808/ECOG 4588^[1]^、RTOG 9410^[2]^、INT0139^[3]^和Furuse^[4]^等多项随机临床研究均证明：同时放化疗治疗局部晚期非小细胞肺癌（locally advanced nonsmall cell lung cancer, LANSCLC）的疗效均优于序贯治疗，5年生存率也相应由单纯放疗时的5%提高到20%以上，中国抗癌协会肺癌专业委员会也提倡有条件的医院可开展同时放化疗的研究。但在放疗同时给予化疗的剂量应当是小剂量还是标准剂量还存在争论，多次小剂量给药的优点是能够提高肺癌放疗的敏感性，同时患者的耐受性相对较好。已有研究^[5-7]^证实紫杉醇可增加细胞有丝分裂期微管的稳定性，染色体不能相互分离，引起G_2_-M期阻滞，进而细胞在分裂期死亡，同时G_2_-M期细胞比例的增高将提高细胞对射线的敏感性。国外Chen等^[8]^研究显示放疗联合小剂量紫杉醇治疗肺癌有效率接近100%。国内朱广迎等^[9, 10]^报道了诺维本、顺铂诱导化疗2个-3个周期后的25例患者采用立体定向适形放疗同步小剂量紫杉醇15 mg/m^2^，3次/周，共6周方案，随访发现副反应轻微，中位生存期23个月，5年生存率为25%，优于国外类似研究^[11]^的疗效，因此研究者推测小剂量化疗合并同时放疗治疗肺癌可能是一种高效、低毒的治疗方法，然而紫杉醇每周三次用法（15 mg/m^2^，3次/周，6周共计270 mg/ m2）与周用法（45 mg/m^2^，1次/周，6周共计270 mg/m^2^）的优劣尚不明确，因此本研究拟在全国多中心随机入组符合条件的患者中，以近期疗效、副反应、生存率为主要指标，比较两种用法的优劣，以优选放疗中紫杉醇的最佳给药方案。

## 1 材料与方法

### 1.1 病例入选标准

多中心随机入组经病理证实不能手术的Ⅲa-Ⅲb NSCLC患者；有可测量的病灶，年龄18岁-70岁，性别不限，6个月内体重下降少于10%，能耐受放疗，预计生存期≥12个月；身体状况评分0分-1分；受试者无主要器官的功能障碍，血常规、肝、肾功能及心脏功能基本正常，实验室化验指标必须符合下列要求：血液：白细胞>4.0×10^9^/L、嗜中性粒细胞≥1.5×10^9^/L、血小板>100×10^9^/L、血红蛋白>95 g/L，血肌苷≤1.5×正常上限。肝功能：总胆红素≤1.5×正常上限，谷丙转氨酶、谷草转氨酶和乳酸脱氢酶≤1.5×正常上限，碱性磷酸酶≤5×正常上限；第一秒用力呼气量（forced expiratory volume in one second, FEV1）≥1 L并且>50%正常人相应值；既往无放疗史，近4周内未接受其它抗肿瘤治疗，已接受全身化疗的周期数≤4（原发灶直径>8 cm应先诱导化疗2个-4个周期）；理解本研究的情况并签署知情同意书。

### 1.2 病例排除标准

入组前接受全身化疗>5个周期化疗者；其它部位恶性肿瘤病史，不包括可治愈的非黑色素性皮肤癌和子宫颈原位癌；妊娠、哺乳期患者；目前存在感染，但感染控制后可以入组；不可控制的糖尿病—任意血糖>13.1 mol/L，但如果患者任意血糖>13.1 mol/ L，空腹血糖 < 9.17 mol/L，该患者可入组；有明确的紫杉醇过敏史；肝、肾功能不全者；其它严重疾病，如6个月内发生过心肌梗塞；患有不易控制的精神病史者。

### 1.3 退出标准

紫杉醇治疗后出现过敏反应；受试者本人要求退出试验；病情进展；任何原因推迟治疗2周以上。

### 1.4 研究方案

#### 1.4.1 试验分组及随机方法

A组为紫杉醇每周三次用法即15 mg/m^2^，3次/周，6周共计270 mg/m^2^；B组为紫杉醇周用法即45 mg/m^2^/周，6周共计270 mg/m^2^。随机方法采用SPSS 13.0软件生成的随机数字表。

#### 1.4.2 诱导化疗方案

患者入组前接受诱导化疗不超过4个周期，诱导化疗方案为NSCLC一线化疗方案即TP（紫杉醇/顺铂）、TC（紫杉醇/卡铂）、NP（长春瑞滨/顺铂）、GP（吉西他滨/顺铂）方案。

#### 1.4.3 同步化疗方案及紫杉醇用法

A组：15 mg/m^2^紫杉醇溶于体积分数为5%葡萄糖250 mL（或0.9%氯化钠注射液）静脉滴注，每周一、三、五早晨8:30开始，静滴1 h，使用紫杉醇前0.5 h静脉注射地塞米松5 mg，肌肉注射苯海拉明40 mg-50 mg，静脉注射西米替丁300 mg-400 mg，化疗前15 min给予止吐药。大约1 h完成紫杉醇输注，至少间隔4 h开始放疗；B组：45 mg/m^2^紫杉醇溶于体积分数为5%葡萄糖（或0.9%氯化钠注射液）250 mL静脉滴注，每周一早晨8:30开始，静滴1 h，使用紫杉醇前预处理同A组。

### 1.5 适形放疗方案及要求

#### 1.5.1 定位CT扫描

采用团注造影剂增强CT，层厚5 mm，层距5 mm。扫描范围为环状软骨上缘至肝脏下缘。

#### 1.5.2 靶区勾划

肿瘤靶区（gross tumor volume, GTV）：包括CT上显示的原发肿瘤、短径>1 cm或PET上标准摄取值（standard uptake value, SUV）>2.5的淋巴结，原发肿瘤区在肺窗中勾画，纵隔病变则在纵隔窗勾画；根据模拟机下肿瘤运动情况确定呼吸运动幅度；计划靶区（lanning target volume, PTV）=GTV+呼吸运动+摆位误差。主管医生可以根据靶区周围重要器官情况适当修改靶区。如果患者有梗阻性肺不张，治疗中每2周胸透或胸片观察复张情况，及时进行再次定位和计划设计。

#### 1.5.3 放疗计划的设计和要求

三维适形或调强放疗，95%PTV单次剂量为（1.8-2.0）Gy/次，共（60-70）Gy/（30-35）次/（6-7）周，45 Gy-50 Gy时缩野做二次改野计划。短径>1 cm的锁骨上淋巴结局部照射相同总剂量。常规做肺校正，要求V20 < 30%，脊髓最大剂量（1 cm^3^） < 40 Gy。

### 1.6 副作用及近期疗效评价

结束时按WHO肿瘤疗效近期疗效评价标准和美国NCI制定的常用毒性评价标准（CTC3.0）评价近期疗效和副反应。

### 1.7 随访及统计学处理

所有入组患者从入组开始定期随访，2年内每3个月一次，2年-5年内每6个月一次。生存期定义为患者放疗开始日期至最后随访日期或死亡日期。统计学软件采用SPSS 13.0，统计学方法采用卡方检验、寿命表法及*Kaplan-Meier*法，*P* < 0.05为有统计学差异。

## 2 结果

### 2.1 入组患者一般资料

2006年4月-2009年4月，入组38例LANSCLC患者。每周三次用法组男性13例，女性5例，中位年龄58岁（27岁-70岁），ECOG评分0分-1分，4例为Ⅲa期，14例为Ⅲb期，其中鳞癌8例，腺癌8例，大细胞癌2例。每周一次用法组男性16例，女性4例，中位年龄59.5岁（45岁-70岁），ECOG评分0分-1分，5例为Ⅲa期，15例为Ⅲb期，其中鳞癌11例，腺癌8例，大细胞癌1例。所有患者入组前签署了知情同意书，详细情况见表 1。

**1:** 38例入组患者一般资料 The baseline characteristics of 38 enrolled patients

Characteristic		No.of patients		*P*
		Group A	Group B	
Age (year)	Median	58	59.5	
	Range	27-70	45-70	
Sex	Male	13	16	0.856
	Female	5	4	
Race	Han	17	19	1.000
	Other	1	1	
ECOG PS	0	12	14	0.825
	1	6	6	
Weight loss	≤5%	16	15	0.494
	>5%	2	5	
Smoking history		10	12	0.822
Forced expiratory volume in one second ≥1 L		18	20	0.842
Induction chemotherapy	Paclitaxel/Cisplatin/carboplatin	7	8	0.891
	Vinorelbine/Cisplatin	5	5	
	Gemcitabine/Cisplatin	6	7	
Stage	Ⅲa	4	5	1.000
	Ⅲb	14	15	
Histology	Squamous cell carcinoma	8	11	0.701
	Adenocarcinoma	8	8	
	Adenosquamous	2	1	
Group A: paclitaxel 15 mg/m^2^, 3 times/week; Group B: paclitaxel 45 mg/m^2^, 1 time/week.				

### 2.2 患者治疗相关副反应

每周三次用法组，1例治疗结束后1年出现3度放射性肺损伤，后因肺内感染呼吸衰竭死亡，3例患者出现1度-2度放射性肺炎，1例患者出现3度放射性食道炎，12例患者出现1度-2度放射性食道炎，13例患者出现1度-2度白细胞减少；每周一次用法组，3例患者出现3度放射性肺炎，3例患者出现2度放射性肺炎，经过激素、抗感染、吸氧、维生素等处理治愈，1例患者出现3度放射性食道炎，10例患者出现1度-2度放射性食道炎，1例患者出现4度白细胞减少，14例患者出现1度-3度白细胞减少。两组患者的血液毒性见表 2、非血液毒性见表 3。

**2:** 38例入组患者的非血液毒性结果 Nonhemotological toxicity of 38 enrolled patients

Group	*n*	Grade (NCI toxicity criteria)																																											
		Weight loss					Fatigue					Fever					Esophagitis					Nausea					Vomiting					Cough					Dyspnea					Pneumonitis			
		1	2	3	4		1	2	3	4		1	2	3	4		1	2	3	4		1	2	3	4		1	2	3	4		1	2	3	4		1	2	3	4		1	2	3	4
A	18	2	-	-	-		3	1	-	-		2	1	-	-		10	2	1	-		1	-	-	-		-	-	-	-		1	1	-	-		1	-	1	-		2	1	1	-
B	20	5	-	-	-		3	3	2	-		2	5	-	-		7	3	1	-		2	-	-	-		-	-	-	-		3	4	1	-		2	4	1	-		-	3	3	-
There was no difference between two groups.																																													

**3:** 38例入组患者的血液毒性结果 Hemotological toxicity of 38 included patients

Group	*n*	Grade (NCI toxicity criteria)																		
		Leukocytes					Neutrophils					Hemoglobin					Platelets			
		1	2	3	4		1	2	3	4		1	2	3	4		1	2	3	4
A	18	9	4	-	-		3	1	-	-		9	1	-	-		1	1	-	-
B	20	5	8	5	1		5	6	3	-		8	4	-	-		1	3	-	-
There was no difference between two groups.																				

### 2.3 患者近期疗效

每周三次用法组共18例患者，完全缓解1例，部分缓解15例，稳定1例，进展1例，患者治疗期间出现脑转移，有效率88.8%；每周一次用法组共20例，完全缓解1例，部分缓解9例，稳定9例，进展1例，患者治疗期间出现脑转移，有效率50.0%。两组近期疗效有统计学差异（*P*=0.026），详细情况见表 4。

**4:** 38例入组患者的近期疗效结果 Response to treatment of 38 enrolled patients

	Group A	Group B	*P*
Complete response (CR)	1	1	
Partial response (PR)	15	9	
Stable disease (SD)	1	9	
Progression	1	1	
Objective response (CR+PR)	88.9%	50%	0.026

### 2.4 患者的生存

所有患者末次随访时间为2009年8月12日，中位随访时间12.0个月（3.0个月-38.0个月），每周三次用法组死亡3例，每周次一用法组死亡6例，除了每周三次用法组1例患者死于放射性损伤合并肺感染外，余患者均死于肿瘤进展，两组患者1年生存率分别为79%和67%，无统计学差异（*P*=0.607）。两组患者生存情况详见图 1。

**1:**
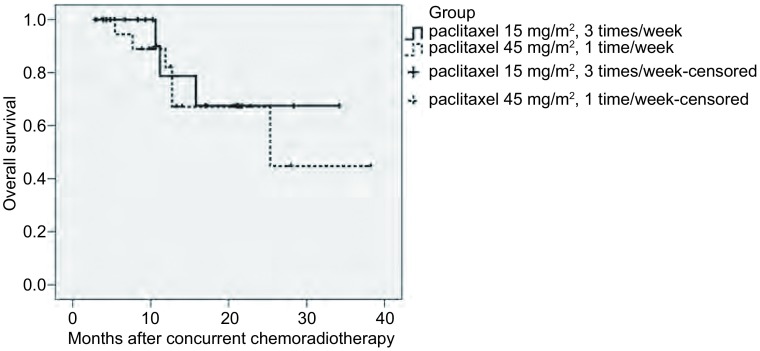
138例入组患者的生存曲线。两组患者1年生存率分别为79%和67%（*P*=0.607） Overall survival in 38 enrolled patients treated with induction chemotherapy and concurrent radiotherapy and paclitaxel. 1-year survival rate of two groups was 79% and 67%, respectively (*P*=0.607)

## 3 讨论

紫杉醇放射增敏作用的机制尚未完全阐明，除了细胞周期阻滞、诱导凋亡外，还与促进乏氧肿瘤细胞再氧合、抑制细胞增殖有关^[12]^。药物浓度、用药和放疗之间的时间间隔也是其影响因素^[13]^。国外已有放疗联合紫杉醇不同剂量方案治疗NSCLC的报道^[14-17]^，用药方案主要包括持续灌注、每周一次和每周二次等，所报道的疗效和毒性差别也较大，因此，放疗联合紫杉醇治疗肺癌的最佳模式仍有争议。

根据Chen^[8]^以及我们的前期研究^[9]^结果，设计LANSCLC同步放化疗中紫杉醇两种用法多中心、随机对照研究。研究结果表明：紫杉醇每周三次用法组18例患者近期有效率88.8%，与国内外报告^[10, 14]^的结果相近，仅1例出现2度放射性肺炎，1例治疗结束后1年出现3度放射性肺损伤，13例患者出现1度-2度放射性食道炎，这与我们以前的研究^[9, 10]^结果一致，毒性低于Chen^[8]^的研究结果，考虑可能是因为后者研究中有两组患者紫杉醇用药剂量高于本研究，分别为20 mg/m^2^、25 mg/m^2^（每周三次），毒性与化疗剂量相关，而且Chen^[8]^的研究结果提示20 mg/m^2^、25 mg/m^2^（每周三次）组与15 mg/m^2^（每周三次）组近期疗效相似。而紫杉醇每周用法组20例患者近期有效率仅50.0%，有效率低于每周三次用法，考虑可能原因是阻滞在G_2_-M期的细胞、乏氧肿瘤细胞再氧合的比例低于每周三次用法组^[12, 13]^。紫杉醇每周用法组较每周三次用法组毒性明显，3例患者出现3度放射性肺炎，发生率15%，11例患者出现1度-3度放射性食道炎，发生率55.5%，1例患者出现4度骨髓抑制，发生率5%，这与文献^[16, 17]^报道相似，毒性增加主要考虑可能与紫杉醇单次化疗剂量强度增加相关。尽管该研究初步结果提示紫杉醇每周三次用法组在毒性、近期疗效方面优于每周用法组，但二者1年生存率并无统计学差异，考虑与样本量较小及随访时间短有关。

总之，该研究初步结果显示诱导化疗后放疗同时合并紫杉醇每周三次用法较每周一次用法治疗LANSCLC安全、有效。由于本研究样本量较小、患者随访时间短，因此尚不知紫杉醇每周三次用法在生存方面是否优于每周一次用法，因此有必要进一步扩大样本量、继续随访患者，以优选放疗中紫杉醇的最佳给药方案。
